# Changes in the Brain-Derived Neurotrophic Factor Are Associated with Improvements in Diabetes Risk Factors after Exercise Training in Adolescents with Obesity: The HEARTY Randomized Controlled Trial

**DOI:** 10.1155/2018/7169583

**Published:** 2018-09-30

**Authors:** Jeremy J. Walsh, Amedeo D'Angiulli, Jameason D. Cameron, Ronald J. Sigal, Glen P. Kenny, Martin Holcik, Steve Doucette, Angela S. Alberga, Denis Prud'homme, Stasia Hadjiyannakis, Katie Gunnell, Gary S. Goldfield

**Affiliations:** ^1^Healthy Active Living and Obesity Research Group, Children's Hospital of Eastern Ontario Research Institute, Ottawa, ON, Canada; ^2^Department of Neuroscience, Carleton University, Ottawa, ON, Canada; ^3^School of Human Kinetics, University of Ottawa, Ottawa, ON, Canada; ^4^Clinical Epidemiology Program, Ottawa Hospital Research Institute, Ottawa, ON, Canada; ^5^Departments of Medicine, Cardiac Sciences and Community Health Sciences, Cumming School of Medicine, University of Calgary, Calgary, AB, Canada; ^6^Department of Health Sciences, Carleton University, Ottawa, ON, Canada; ^7^Department of Community Health & Epidemiology, Dalhousie University, Halifax, NS, Canada; ^8^Department of Exercise Science, Concordia University, Montreal, QC, Canada; ^9^Institut du Savoir Montfort, Ottawa, ON, Canada; ^10^Centre for Healthy Active Living, Children's Hospital of Eastern Ontario, Ottawa, ON, Canada; ^11^Department of Psychology, Carleton University, Ottawa, ON, Canada; ^12^Department of Pediatrics, University of Ottawa, Ottawa, ON, Canada; ^13^School of Psychology, University of Ottawa, Ottawa, ON, Canada

## Abstract

Obesity in youth increases the risk of type 2 diabetes (T2D), and both are risk factors for neurocognitive deficits. Exercise attenuates the risk of obesity and T2D while improving cognitive function. In adults, these benefits are associated with the actions of the brain-derived neurotrophic factor (BDNF), a protein critical in modulating neuroplasticity, glucose regulation, fat oxidation, and appetite regulation in adults. However, little research exists in youth. This study examined the associations between changes in diabetes risk factors and changes in BDNF levels after 6 months of exercise training in adolescents with obesity. The sample consisted of 202 postpubertal adolescents with obesity (70% females) aged 14–18 years who were randomized to 6 months of aerobic and/or resistance training or nonexercise control. All participants received a healthy eating plan designed to induce a 250/kcal deficit per day. Resting serum BDNF levels and diabetes risk factors, such as fasting glucose, insulin, homeostasis model assessment (HOMA-B—beta cell insulin secretory capacity) and (HOMA-IS—insulin sensitivity), and hemoglobin A1c (HbA1c), were measured after an overnight fast at baseline and 6 months. There were no significant intergroup differences on changes in BDNF or diabetes risk factors. In the exercise group, increases in BDNF were associated with reductions in fasting glucose (*β* = −6.57, SE = 3.37, *p* = 0.05) and increases in HOMA-B (*β* = 0.093, SE = 0.03, *p* = 0.004) after controlling for confounders. No associations were found between changes in diabetes risk factors and BDNF in controls. In conclusion, exercise-induced reductions in some diabetes risk factors were associated with increases in BDNF in adolescents with obesity, suggesting that exercise training may be an effective strategy to promote metabolic health and increases in BDNF, a protein favoring neuroplasticity. This trial is registered with ClinicalTrials.gov NCT00195858, September 12, 2005 (funded by the Canadian Institutes of Health Research).

## 1. Introduction

Pediatric obesity currently affects 13–20% of children and youth in Westernized countries [1, 2]. Obesity is associated with a myriad of deleterious health outcomes, including diminished neurocognitive function in childhood [3], an increased risk of Alzheimer's disease, and dementia later in life [4], possibly due to increased inflammatory cytokines which have systemic effects linked to glycemic dysregulation and the initiation of type 2 diabetes (T2D) pathology [5]. Obesity is well known to increase the risk of T2D [6], and T2D in adulthood is one of the strongest independent risk factors for neurological disease [7, 8]. In children and youth, recurrent hyperglycemia is associated with adverse structural brain outcomes, including reduced gray and white matters [9], suggesting that improving metabolic regulation early in life is important for long-term brain health. Although poorly understood, the proposed mechanisms contributing to increased risk of neurocognitive dysfunction with these conditions include poor glycemic regulation [10] and alterations in the brain-derived neurotrophic factor (BDNF) [11].

BDNF is a member of the neurotrophin family of proteins and is chiefly recognized for its role in facilitating aspects of neural plasticity and long-term potentiation [12]. In addition to its neurogenic effects, BDNF also exerts metabotropic effects, including the regulation of whole-body energy homeostasis and feeding behavior [11], skeletal muscle fat oxidation [13], beta cell functioning [14], and hepatic glycemic control [14]. BDNF crosses the blood-brain barrier in a bidirectional manner [15], and circulating levels reflect cortical BDNF [16, 17]. Low circulating BDNF is associated with both neurological and metabolic conditions including, major depressive disorder [18], Alzheimer's disease [19], obesity [20, 21], and T2D [22]. In fact, adults with obesity and adults with T2D have significantly lower circulating BDNF compared to lean, nondiabetic adults, possibly owing to hyperglycemia-induced reductions in plasma BDNF [22]. It has recently been suggested that BDNF may play an etiological role in obesity and metabolic syndrome via *in utero* “developmental programming” of the obesity and T2D phenotypes [23]. Indeed, BDNF-haploinsufficient mice are obese and diabetic and exhibit hyperphagic behavior [24], and expression of the *BDNF* gene variant (Val66Met) is significantly associated with measures of obesity in human pediatric [20] and adult cohorts [21].

Interestingly, increasing BDNF levels improves metabolic regulation, as intracerebroventricular infusion of exogenous BDNF suppresses appetite [24, 25] and improves systemic glycemic control via hepatocyte insulin sensitivity [26], pancreatic beta cell function [14, 27], and increased glucose utilization [28] in animal models. Further, overexpression of hypothalamic BDNF in mice facilitates the browning of adipose tissue via sympathetic nerve activity, leading to increased energy expenditure and reduced plasma glucose levels [29, 30]. Collectively, these findings demonstrate the important role of BDNF in metabolic regulation. Therefore, interventions that increase BDNF expression may have favorable effects on metabolic function while improving neurocognitive outcomes in youth with obesity and/or T2D [23, 31].

Exercise training is one intervention strategy that could both target BDNF and improve metabolic function. Exercise plays an important role in the management of pediatric obesity [32] and its metabolic comorbidities [33] and is also a potent tool for increasing circulating levels of BDNF [34, 35]. Acute bouts of moderate-to-vigorous intensity aerobic exercise consistently increase circulating BDNF in adults [35], and aerobic exercise training of at least two weeks in duration generally, but not universally, increases resting BDNF levels in adults [34, 36]. Jeon and Ha [37] found that 12 weeks of high-intensity aerobic training increases resting serum BDNF in healthy adolescent males, which corresponded with neurocognitive (working memory) improvements. Lee et al. [38] observed a 2.5-fold increase in resting serum BDNF and ~6% reduction in adiposity following 12 weeks of moderate-intensity aerobic training in adolescents with obesity, whereas these changes were not observed in those with T2D or in healthy-weight controls. Although these findings suggest that exercise may disproportionately increase BDNF in youth with obesity compared to youth with T2D, the small sample size (*n* = 26 overall), the high variability in the BDNF measures, and the sample composition of only males warrant caution in drawing conclusions from this work. Moreover, no indicators of glucose regulation were reported, thus precluding an evaluation of how changes in BDNF relate to changes in diabetes risk in the adolescents with obesity.

Given the involvement of BDNF in metabolic regulation and the ability of exercise to increase BDNF and to improve body composition and metabolic outcomes, it stands to reason that changes in diabetes risk factors and adiposity may be related to changes in BDNF following an exercise intervention in youth with obesity. However, no study to date has investigated these relationships in this population. The primary aim of this study was to quantify the independent contribution that demographic, anthropometric, and metabolic indicators make toward the prediction of change in serum BDNF levels after a 6-month exercise intervention among a high-risk population of adolescents with obesity. Given the mechanistic evidence from animal models, we hypothesized that changes in metabolic indicators including adiposity, circulating glucose and insulin, insulin sensitivity and beta cell function, and hemoglobin A1c (HbA1c) would be associated with changes in BDNF levels from exercise training. This study represents secondary data analysis from the healthy eating, aerobic, and resistance training in youth (HEARTY) trial [39].

## 2. Materials and Methods

### 2.1. Participants

For this secondary analytic study, we only obtained baseline and postintervention data on resting serum BDNF and diabetes risk factors on 202 of the 304 participants who entered the study, due to availability of blood samples. The sample breakdown for the present study included those who received exercise interventions (*n* = 152) or diet-only controls (*n* = 50). Participants included 66 boys and 136 girls aged 14–18 years (15.4 yrs ± 1.4), who volunteered for the HEARTY trial conducted in Ottawa, Canada.

#### 2.1.1. Recruitment

Inclusion criteria were inactive postpubertal (Tanner stages IV–V) adolescents aged 14–18 years with BMI > 95^th^ percentile for age/sex or ≥85^th^ percentile for age/sex with an additional diabetes or cardiovascular disease risk factor as described elsewhere [40]. Exclusion criteria were any regular physical activity (more than twice a week for more than 20-minute sessions) four months prior, diagnosis of T2D, use of any performance enhancing medication, significant weight change (increase or decrease of ≥5% during the two months before enrollment), pregnancy at the start of the study or intention to become pregnant in the next year, and any contraindications to activity due to disease or other illness (eating disorders/clinical depression) judged by the participant or study physician to make participation inadvisable. A parent or guardian was asked to cosign the consent form of any participant below the age of 16 years. This study received approval from the Research Ethics Boards at the Children's Hospital of Eastern Ontario (protocol #05/04E) and the Ottawa Hospital Research Institute (protocol #2004219-01H). The trial began in March 2005 and was completed in June 2011.

### 2.2. Design and Procedures

This study represents a secondary analysis from an exercise intervention that evaluated the effects of exercise modality on body composition [40]. After completing baseline testing, participants entered a run-in period including supervised exercise training four times weekly for four weeks, whereby participants engaged in low-intensity, low-volume resistance, and aerobic training. To qualify for randomization, participants needed to attend at least 13 of 16 prescribed exercise sessions (>80% adherence) during this run-in phase. Participants (*n* = 202) were randomized to exercise or control groups using central randomization. Participants in the exercise groups were asked to attend gyms 4 times weekly, following exercise programs as previously described protocols [39, 40]. Outcome assessors were blinded to group assignment, ensuring unbiased measurement. As well, all participants received counseling by a registered dietitian that was designed to promote healthy eating with a daily energy deficit of 250 kcal. (see [40], for complete methodological details concerning the HEARTY trial).

### 2.3. Measurements

#### 2.3.1. Demographic and Anthropometric Variables

At baseline, background information was obtained from all participants, including demographics (age, race, ethnicity, and parental education), self-reported pubertal status, measured height and weight for body mass index (BMI), self-reported physical activity, family medical history, and current medical status and medications to evaluate inclusion criteria. BMI was calculated as weight in kilograms divided by height in meters squared. At baseline and 6 months, height and weight were recorded with a manual stadiometer and scale, respectively, with participants wearing light clothing and no shoes. Body composition was assessed by magnetic resonance imaging (MRI) (Echospeed, signal 11 version; GE Medical Systems) at baseline and 6 months. Percent body fat was analyzed using SliceOMatic™ software V.4.3 (TomoVision, Magog, Canada).

#### 2.3.2. Diabetes Risk Factors and BDNF

Twelve-hour (overnight-fasting) samples of approximately 20 mL of venous blood were taken in the morning, from a forearm or antecubital vein, and were stored in a freezer at −80°C. Samples were obtained at baseline before the run-in period and at least 48 hours after the last exercise session at the end of the 6-month intervention to avoid potentially confounding acute effects of exercise. Samples were analyzed for serum BDNF and metabolic indicators, including fasting glucose (mmol/L), fasting insulin (mmol/L), and HbA1c (percentage) as reported by Alberga et al. [40]. The homeostasis model assessment (HOMA) was used to assess beta cell insulin secretory capacity (HOMA-B) and insulin sensitivity (HOMA-IS) using the computer program and equations calculated by Wallace et al. [41] and Levy et al. [42]. HOMA assesses insulin secretory capacity and insulin resistance based on fasting glucose and insulin concentrations from blood samples drawn at the same time. The model is based on data from physiological studies and has been validated against other methods of assessing insulin secretory capacity and insulin [41, 42]. Importantly, HOMA can be used to longitudinally track changes in insulin sensitivity and beta cell function following an exercise intervention [41]. A free, online calculator provided courtesy of the University of Oxford was used to calculate HOMA using HOMA model 2 (https://www.dtu.ox.ac.uk/homacalculator) [41].

BDNF was analyzed using a commercially available ELISA kit (Human Free BDNF Quantikine ELISA kit, R&D Systems, Cat# DBD00) in accordance with the manufacturer's instructions. Serum samples of BDNF were diluted 75-fold into Calibrator Diluent RD6P prior to starting the assay, and all samples were run in duplicate. The sensitivity of this assay product is 20 pg/mL with a range of 62.5–4000 pg/mL. All standard curves were linear within the range used for the analysis with a strong corresponding correlation of coefficient (*r*^2^ > 0.99).

### 2.4. Statistical Analysis

Baseline demographics were summarized as means and standard deviations, and between group differences were examined using a one-way analyses of variance for continuous data. Categorical data were summarized as frequencies and percentages, and group differences at baseline were examined using chi-square tests. Spearman correlations were performed to determine the unadjusted association between changes in body composition, metabolic risk factors, and changes in BDNF levels. We previously ran linear mixed-effects regression modeling for repeated measures to assess the effects of exercise training on changes in resting serum BDNF over time, with BDNF as the dependent variable, group (aerobic, resistance, combined, and control), time (baseline and 6 months), and group × time interaction as the independent variables, with adjustment for age and sex using an unstructured covariance matrix for the repeated measures [43]. Results from these mixed-model regressions and Spearman correlations showed that changes in BDNF levels did not differ significantly by the exercise group [43], and the pattern of association between changes in body composition, metabolic markers, and BDNF was comparable across exercise groups (data not reported). Thus, a combined analytic approach was used whereby all exercise groups were pooled to form one group. Multiple linear regression models were then conducted to identify the independent prediction of changes in BDNF (dependent variable) from changes in anthropometrics and all metabolic indicators. Given that anthropometric variables were colinear, only BMI was used in the regression models to make results comparable to existing anthropometry literature. Moreover, the patterns of association remained the same when other indicators of anthropometry, such as percent body fat and waist circumference, were used. All regression analyses were adjusted for age, gender, and highest level of parent education as a proxy for sociodemographic status. Data are presented as standardized beta weights and standard errors (SEs). Statistical significance was defined as a two-tailed *p* < 0.05. Analyses were conducted using SAS, version 9.4 (SAS Institute, Cary, North Carolina).

## 3. Results

Table 1 shows baseline characteristics of the sample. Most participants (182 of 202 or 90%) had obesity (BMI ≥ 95^th^ percentile for age and sex). The sample was about 70% Caucasian, and most parents had some postsecondary education. There were no statistically significant group differences on sociodemographic variables, body composition, BDNF levels, or diabetes risk factors.

In the main outcome analysis, there was no significant group × time interaction for BDNF (*F*(3, 227) = 0.89, *p* = 0.45), indicating that changes between groups over time did not differ significantly as reported elsewhere [43]. Similarly, there were no group differences in changes in diabetes risk factors from exercise intervention, as reported elsewhere [39]. Gender did not moderate the effects of exercise training on changes in BDNF or diabetes risk factors, as reported elsewhere [39, 43].

There were no significant associations at baseline between BDNF levels and age (*r* = −0.02, *p* = 0.79), gender (*r* = 0.07, *p* = 0.37), highest parent education (*r* = 0.02, *p* = 0.77), BMI (*r* = −0.08, *p* = 0.33), waist circumference (*r* = 0.033, *p* = 0.68), or percent body fat (*r* = 0.00, *p* = 0.99).

Table 2 presents the unadjusted correlations between changes in anthropometrics and diabetes risk factors after 6 months of exercise intervention for all exercise groups combined and controls. As expected, for diet-only controls, no significant correlations emerged between changes in BDNF and changes in fasting glucose (*r* = −0.018, *p* = 0.90), fasting insulin (*r* = −0.043, *p* = 0.77), HbA1c (*r* = −0.015, *p* = 0.92), HOMA-B (*r* = −0.056, *p* = 0.71), and HOMA-IS (*r* = 0.06, *p* = 0.67). In the exercise group, however, unadjusted increases in BDNF were associated with reductions in fasting glucose (*r* = −0.17, *p* = 0.04) and increases in HOMA-B (*r* = 0.23, *p* = 0.005). No significant associations emerged for changes in BDNF and changes in fasting insulin, HbA1c, and HOMA-IS or anthropometrics when all exercise groups were combined.

Table 3 presents the associations between changes in metabolic indicators and anthropometrics and changes in BDNF levels, controlling for age, gender, and highest level of parent education. The associations between 6-month changes in BDNF and changes in fasting glucose (*β* = 6.57, SE = 3.37, *p* = 0.05) and HOMA-B (*β* = 0.093, SE = 0.03, *p* = 0.004) in the exercise group remained statistically significant after adjusting for confounders.

## 4. Discussion

This study investigated the independent associations between changes in serum BDNF, diabetes risk factors, and body composition following a 6-month exercise intervention in adolescents with obesity. The main findings from this study were (a) changes in some diabetes risk factors, most notably fasting glucose and beta cell insulin secretory capacity (HOMA-B), were stronger independent predictors of exercise-induced changes in BDNF than changes in body composition and (b) there was no relationship between anthropometric variables and BDNF at baseline or following the 6-month intervention period.

In animal models, the relationship between obesity and BDNF is quite robust and consistent [23, 30]. The arcuate nucleus of the hypothalamus (ARC) regulates feeding behavior and long-term energy stores of the body (i.e., adiposity) via leptin signaling and glucose sensing [30]. BDNF is thought to mediate neural plasticity in ARC pathways in response to energetic challenges (fasting, caloric restriction) and physical stimulation (physical activity, environmental enrichment), leading to appetite suppression and increased energy expenditure [25, 30]. Downregulation of BDNF and its receptor tropomyosin receptor kinase B (TrkB) in the hypothalamus significantly increases body weight and reduces body temperature and locomotion in rodents [24, 44], whereas the infusion of exogenous BDNF appears to reverse this phenotype [24]. The relationship between whole-body energy stores—particularly adiposity—and BDNF is less consistent in humans, especially in children and youth. In the current study, we did not observe any relationships between adiposity and BDNF levels, which is consistent with a recent sample of 447 healthy adolescents [45], whereas other studies have reported BDNF levels that are both lower [46] or higher [47] in youth with obesity compared to those without. Interestingly, Roth et al. [47] observed that changes in serum BDNF were significantly correlated with changes in adiposity and leptin following a one-year lifestyle intervention targeting weight loss in adolescents with obesity, which appears to be consistent with the relationships found in animal models [30]. Collectively, these findings indicate a complex and somewhat inconsistent relationship between adiposity and resting BDNF in humans, particularly in children, and therefore warrants future inquiry to clarify these relationships in youth.

A major finding of this study was that exercise-induced changes in serum BDNF were significantly associated with improvements in fasting glucose and HOMA-B, a measure of pancreatic beta cell insulin secretion response to a glucose load. From a clinical perspective, this is important given that HOMA-B is one of the strongest risk/protective factors in the development of T2D [48]. Although no directly comparable studies exist in children, the few studies conducted can be informative. Similar to our findings, Araya et al. [49] showed that weight loss from caloric restriction increases serum BDNF levels and improves glucose regulation in adults with overweight and obesity; however, correlations between these measures were not assessed. Conversely, Swift et al. [50] failed to see any relationships between serum BDNF and glucose control following a 9-month exercise intervention in adults with T2D. Similarly, Roth et al. [47] showed that changes in BDNF were not associated with HOMA-insulin resistance; however, we are unable to compare these findings to the current study given that measures of HOMA-B and HOMA-IS were not included. Rodent studies have established the relationship between glucose regulation and BDNF, and combined with some human models, these studies suggest the existence of a bidirectional relationship. Acute hyperglycemia downregulates circulating plasma BDNF via reduced output from the brain in healthy adults, and adults with T2D appear to have lower resting BDNF levels, possibly due to chronic hyperglycemia [22]. Conversely, both repeated and single subcutaneous doses of exogenous BDNF rapidly enhance insulin signaling [26] and lower blood glucose through increases in glucose utilization [28] in the liver, skeletal muscle, and brown adipose tissue of diabetic mice. The hypoglycemic effects of BDNF appear to be mediated through the direct actions of BDNF on target tissue, as well as its modulation of the central and peripheral nervous systems [28]. For example, overexpression of hypothalamic BDNF stimulates the browning of white adipose tissue via sympathetic nerve activity, leading to enhanced energy expenditure and lowering of blood glucose via adaptive thermogenesis [28, 29].

It is well established that acute exercise increases circulating BDNF [35] and may represent a “dose” of BDNF [31], analogous to rodent infusion studies. For instance, 8 weeks of treadmill training increases plasma BDNF in healthy rats and BDNF appears to mediate the exercise-induced improvements in insulin tolerance and increased beta cell size [14, 27]. Accordingly, exercise may improve diabetes risk factors and obesity directly through its effects on glucose regulation and energy expenditure, as well as indirectly via its effects on BDNF and improvements in glucose regulation and metabolic health from exercise. In turn, this may lead to increased BDNF expression, thereby creating a synergistic feedback loop. However, despite the wealth of plausible mechanisms, it is important to note that the exercise-induced changes in the outcome measures of the current study occurred at the same time, thus preluding the determination of temporal sequencing and causal inferences.

This study has limitations and strengths that warrant mention. Strengths include the largest sample size of any randomized controlled trial of supervised exercise in youth that incorporated a broad scope of health indicators and a population with obesity at high risk for both neurocognitive deficits and T2D. It is also the only exercise study in children to include a measure of beta cell function, which led to a novel finding that changes in beta cell function are associated with changes in BDNF following a structured exercise intervention. Limitations of the present study include a sample that was primarily female, Caucasian, and primarily the offspring of well-educated parents; thus, results may not generalize to youth coming from more diverse or disadvantaged sociodemographic backgrounds. These findings may also not be generalizable to children with developmental disabilities, who are at an increased risk of obesity and metabolic disorders [51]. Accordingly, future studies should investigate the relationship between BDNF and metabolic outcomes following programmed exercise interventions in children with developmental disabilities. Longer intervention duration might have allowed a better quantification of trajectories and possible temporal sequencing of changes in BDNF and diabetes risk factors, which should be explored in future research. Future studies should also assess how changes in quality of life and other indicators of psychosocial functioning relate to changes in BDNF and metabolic outcomes in response to an exercise intervention in clinical populations, such as adolescents with obesity. Lastly, although the current study focused solely on the relationship between BDNF and metabolic outcomes in response to exercise training, a noteworthy limitation of this work is the lack of neurocognitive assessment. Inclusion of these measures would provide a more comprehensive understanding of the complex relationships between BDNF, metabolic outcomes, and cognition in response to exercise training in adolescents with obesity. While these limitations cannot be overcome in the present study, our data do provide a foundation to guide selection and evaluation of outcomes for future exercise interventions in the specific population studied, in order to gain a better understanding of the potential neural effects of exercise and their relationship to changes in metabolic outcomes. Developmentally, such relationships seem to be less straightforward to predict than what could be conjectured based on current animal and adult models.

In conclusion, our data show that exercise-induced reductions in selected diabetes risk outcomes were associated with increases in resting serum BDNF levels in adolescents with obesity. Given that BDNF plays a central role in facilitating learning, memory, and neural plasticity [11], combined with obesity representing potent risk factors for both neurocognitive deficits [3] and metabolic disorders [10], our findings coupled with previous studies provide a strong rationale for targeting BDNF signaling via exercise interventions to treat the clustering of obesity, diabetes, and neurological disorders.

## Figures and Tables

**Table 1:** Participant characteristics at baseline.

	Total sample (*n* = 202)	Exercise (*n* = 152)	Control (*n* = 50)
Males, *N* (%)	66 (32.7)	50 (32.9)	16 (32)
Females, *N* (%)	136 (67.3)	102 (67.1)	34 (68)
Age (years)	15.5 (1.4)	15.5 (1.4)	15.4 (1.3)
Weight (kg)	97.8 (17.1)	97.9 (16.7)	97.5 (18.3)
Height (cm)	168.1 (7.6)	168.0 (7.7)	168.3 (7.1)
BMI (m/kg^2^)	34.5 (4.4)	34.5 (4.1)	34.3 (5.1)
Percent body fat	49.1 (5.4)	49.3 (5.3)	48.5 (5.6)
Waist circumference (cm)	96.7 (10.6)	97.1 (10.3)	95.3 (11.2)
BDNF	26 (14.2)	26.2 (14.3)	25.2 (14)
Glucose	5 (0.4)	5.0 (0.4)	5.1 (0.4)
Insulin	103.6 (58)	99.4 (52.0)	116.4 (72.5)
HOMA %B	145.5 (50.4)	144.6 (50.6)	148.5 (50.3)
HOMA %IS	65.9 (30.3)	67.9 (31.3)	59.8 (26.5)
HbA1c (%)	5.0 (0.3)	5.0 (0.3)	5.0 (0.2)
Ethnicity, *N* (%)			
Caucasian	149 (73.8)	107 (70.4)	42 (84.0)
Black	14 (6.9)	12 (7.9)	2 (4.0)
Mixed racial	7 (3.5)	6 (3.9)	1 (2.0)
Arabic	8 (4.0)	7 (4.6)	1 (2.0)
Hispanic	9 (4.5)	9 (5.9)	0 (0.0)
Asian	6 (3.0)	5 (3.3)	1 (2.0)
Other	4 (2.0)	3 (2.0)	1 (2.0)
Native Canadian	4 (2.0)	2 (1.3)	2 (4.0)
Indonesian/Asian	1 (0.5)	1 (0.7)	0 (0.0)
Highest parental education, *N* (%)			
High school or less	30 (14.9)	22 (14.5)	8 (16.0)
College/university	172 (85.1)	130 (85.5)	42 (84.0)

Note: BMI = body mass index; BDNF = brain-derived neurotrophic factor; HbA1c = hemoglobin A1c; HOMA-B = homeostatic model assessment beta cell secretory capacity; HOMA-IS = homeostatic model assessment insulin sensitivity.

**Table 2:** Relationship between changes in BDNF and changes in anthropometrics and diabetes risk factors in the exercise group.

Measure	Correlation, *r*	*p* value
Δ weight	−0.005	0.95
Δ waist circumference	0.02	0.81
Δ BMI	0.01	0.83
Δ %body fat	−0.04	0.6
Δ glucose	−0.17	0.04
Δ insulin	0.13	0.1
Δ HbA1c	0.01	0.89
Δ HOMA-B	0.23	0.005
Δ HOMA-IS	−0.06	0.46

Note: BMI = body mass index; HbA1c = hemoglobin A1c; HOMA-B = homeostatic model assessment beta cell secretory capacity; HOMA-IS = homeostatic model assessment insulin sensitivity.

**Table 3:** Changes in diabetes risk factors predicting change in BDNF in the exercise group.

Effect	Beta estimate	SE	*p* value
Change in glucose	−6.565	3.374	0.054
Age	−0.176	1.028	0.864
Gender	2.240	3.105	0.472
Highest parental education	0.444	1.856	0.811
Change in BMI	0.185	0.716	0.797
Change in HOMA-B	0.093	0.031	0.004
Age	−0.551	1.031	0.594
Gender	3.802	3.129	0.226
Highest parental education	0.752	1.841	0.683
Change in BMI	−0.652	0.766	0.396
Change in HOMA-IS	−0.025	0.033	0.450
Age	−0.592	1.069	0.581
Gender	3.206	3.247	0.325
Highest parental education	0.785	1.895	0.679
Change in BMI	−0.133	0.787	0.866
Change in HbA1c (per 0.1%)	0.001	0.568	0.999
Age	−0.238	1.035	0.818
Gender	2.563	3.143	0.416
Highest parental education	0.650	1.882	0.730
Change in BMI	0.173	0.750	0.818
Change in insulin	0.049	0.027	0.076
Age	−0.643	1.048	0.540
Gender	3.478	3.209	0.280
Highest parental education	0.951	1.878	0.613
Change in BMI	−0.358	0.773	0.644

Note: BMI = body mass index; HbA1c = hemoglobin A1c; HOMA-B = homeostatic model assessment beta cell secretory capacity; HOMA-IS = homeostatic model assessment insulin sensitivity.

## Data Availability

The data used to support the findings of this study are available from the corresponding author upon request.
